# The Pharmacochaperone Activity of Quinine on Bitter Taste Receptors

**DOI:** 10.1371/journal.pone.0156347

**Published:** 2016-05-25

**Authors:** Jasbir D. Upadhyaya, Raja Chakraborty, Feroz A. Shaik, Appalaraju Jaggupilli, Rajinder P. Bhullar, Prashen Chelikani

**Affiliations:** 1 Department of Oral Biology, and Manitoba Chemosensory Biology (MCSB) Research group, University of Manitoba, Winnipeg, MB, R3E 0W2, Canada; 2 Children’s Hospital Research Institute of Manitoba, Winnipeg, MB, R3E 3P4, Canada; The University of Tokyo, JAPAN

## Abstract

Bitter taste is one of the five basic taste sensations which is mediated by 25 bitter taste receptors (T2Rs) in humans. The mechanism of bitter taste signal transduction is not yet elucidated. The cellular processes underlying T2R desensitization including receptor internalization, trafficking and degradation are yet to be studied. Here, using a combination of molecular and pharmacological techniques we show that T2R4 is not internalized upon agonist treatment. Pretreatment with bitter agonist quinine led to a reduction in subsequent quinine-mediated calcium responses to 35 ± 5% compared to the control untreated cells. Interestingly, treatment with different bitter agonists did not cause internalization of T2R4. Instead, quinine treatment led to a 2-fold increase in T2R4 cell surface expression which was sensitive to Brefeldin A, suggesting a novel pharmacochaperone activity of quinine. This phenomenon of chaperone activity of quinine was also observed for T2R7, T2R10, T2R39 and T2R46. Our results suggest that the observed action of quinine for these T2Rs is independent of its agonist activity. This study provides novel insights into the pharmacochaperone activity of quinine and possible mechanism of T2R desensitization, which is of fundamental importance in understanding the mechanism of bitter taste signal transduction.

## Introduction

Bitter taste, in humans, is perceived by a family of 25 G protein-coupled receptors (GPCRs), referred to as T2Rs [1]. These receptors vary in their selectivity for bitter compounds; some receptors are activated by only few substances, whereas, others show a broad ligand spectrum [2–8]. It is now clear that bitter taste signaling is not limited to taste buds, and T2Rs are expressed in many extraoral tissues [9]. They mediate protective reflexes by performing different physiological roles, including bronchodilation, in extraoral tissues and are implicated as potential therapeutic drug targets in the treatment of asthma [10, 11]. Thus, it becomes important to understand how T2R signaling is regulated. GPCRs regulate the strength and duration of signal transduction to adapt to changing external conditions and to avoid damage from sustained signaling. The earliest event in GPCR desensitization is receptor phosphorylation [12]. Two families of kinases are currently known to contribute to the desensitization of GPCRs; second messenger-dependent protein kinase A (PKA) or protein kinase C (PKC), and GPCR-specific G protein-coupled receptor kinases (GRKs) [13].

Though there have been well documented studies on the regulation of GPCR signaling for many receptors such as adrenergic receptor, muscarinic, prostanoid receptors, very little is known about the desensitization and internalization of chemosensory receptors like T2Rs. A previous study showed that like most GPCRs, endogenous T2Rs in human airway smooth muscle (ASM) undergo desensitization upon exposure to quinine and proposed the involvement of GRKs in the process [14]. Therefore this study was designed to characterize the desensitization and internalization of T2R4 upon agonist-stimulation.

Recently we pursued extensive structure-function studies, including mapping of the agonist quinine-binding pocket, on T2R4 [15–19]. Thus, the well-characterized bitter taste receptor T2R4 was selected for this study in which we characterize its desensitization and internalization upon agonist stimulation. Using quinine and yohimbine, we examined the desensitization of T2R4 after transiently expressing it in HEK293T cells. Quinine is an alkaloid isolated from the bark of cinchona tree and is the most intense bitter tasting compound. To monitor the potential non-specific (or heterologous) forms of desensitization, the calcium responses of mock-transfected cells were subtracted from the receptor-specific responses, suggesting a homologous form of receptor desensitization. To analyze agonist-induced receptor internalization, cell surface expression of T2R4, upon exposure to different bitter compounds, was examined. Surprisingly, quinine caused upregulation of T2R4 surface expression. This phenomenon of chaperone activity was also observed in other T2Rs that are activated by quinine. Our results suggest that unlike most other GPCRs, T2Rs do not undergo internalization upon agonist treatment, and quinine acts as a pharmacological chaperone for T2R4, T2R7, T2R10, T2R39 and T2R46.

## Materials and Methods

### Materials

HEK293T cells were obtained from ATCC and maintained in 10% fetal bovine serum at 37°C in a 95% air and 5% CO_2_ chamber. hASMCs were a kind gift from Dr Andrew Halayko, Dept. of Physiology, University of Manitoba [20]. Quinine hydrochloride, yohimbine, denatonium benzoate, dapsone, parthenolide and dextromethorphan hydrobromide (DXM) were purchased from Sigma Aldrich (ON, Canada). Brefeldin A (BFA) was purchased from Cell Signaling Technology (ON, Canada), Mouse monoclonal M2 anti-FLAG antibody was from Sigma Aldrich and rabbit polyclonal anti-T2R4 antibody was from ThermoFisher Scientific (Toronto, ON, Canada). Goat anti-mouse Alexa Fluor-488 and goat anti-rabbit Alexa Fluor-488 were purchased from Molecular Devices (CA, USA). APC conjugated anti-FLAG monoclonal antibody was from BioLegend (CA, USA). The synthetic oligonucleotide primer sequences for human TAS2R4 (F -TCCTGCTGAAGCGGAATATC; R–GAAAAGGTGATGCCTGGCTA) were purchased from Invitrogen.

### Molecular Biology and Cell culture

Human TAS2R4 (untagged), N-terminal FLAG epitope tagged human TAS2R1, TAS2R3, TAS2R4 genes and thromboxane A2 receptor-α isoform (TPα) carried by expression vector pcDNA 3.1/Neo, and TAS2R7, TAS2R10, TAS2R14, TAS2R39, TAS2R40, TAS2R43, TAS2R44 and TAS2R46 carried by expression vector pcDNA 3.1/Hygro (Invitrogen, ON, Canada) were commercially synthesized as previously described [15, 21, 22]. The gene constructs in pcDNA 3.1/Hygro include a FLAG epitope sequence at the 5’ following the ATG start codon. To facilitate cloning, restriction sites for Kpn1 and EcoR1 at 5’and Not1 at 3’ were included. HEK293T cells were cultured in DMEM/F12 media supplemented with 10% fetal bovine serum, penicillin (100 mg/ml) and streptomycin (100 mg/ml). The wild type TAS2R4 or other genes in pcDNA 3.1 were transiently expressed in HEK293T cells using Lipofectamine 2000 (Invitrogen) according to manufacturer’s instructions. Quinine was used at a concentration of 1 mM, which corresponds to its EC_50_ value for T2R4, in all experiments.

### Quantitative PCR

TAS2R4-transfected HEK293T cells in 6-well plate were treated with quinine for 15 min or 1 h. Total RNA was extracted following 48 h of transfection using RNeasy Mini kit (Qiagen). Genomic DNA was removed by treating 10 μg of the extracted RNA with 8 units of DNase I (New England Biolabs) for 75 mins at 37°C. About 1 μg of purified RNA was reverse transcribed using SSIII RT (superscript III reverse transcriptase, Invitrogen), dNTPs, OligodT primer and first strand buffer. The synthesized cDNA was used as template for amplification in PCR. Reaction mixtures with a final volume of 10 μl consisted of reverse transcribed cDNA, primers, 1x SYBR Green containing dNTP mix and Taq polymerase. The reaction consisted of the following steps; an initial denaturation step of 1 min at 95°C then 50 cycles of 94°C for 30 sec, annealing at 58°C for 30 sec and extension at 72°C for 30 sec and a final extension at 72°C for 2 min. This was followed by melt curve analysis from 72°C to 95°C at every 1°C increase in temperature for about 1 sec for 23 cycles. Melt curve analysis confirmed the presence of a single PCR product in each reaction. Analysis was performed by 2^-ΔCt^ method. An illumina real time PCR detection system was used for these experiments.

### Flow cytometry

The cell surface expression of untagged wild type T2R4, FLAG-T2R1, FLAG-T2R3, FLAG-T2R4, and FLAG-TPα was determined using a BD FACS Canto flow cytometer. HEK293T cells, in 6-well tissue culture plate, were transfected with 3 μg of DNA per 1×10^6^ cells. Following 24–36 h of transfection, the cells were incubated with 1 mM each of quinine, yohimbine, dapsone, parthenolide, 2 mM DB, or 1 μM U46619, a TPα agonist, at 37°C for 1 h or for the indicated time points. The hASMCs used for determining cell surface expression of endogenous T2R4 were from passage P2 or P3. Viable cells at a concentration of 1×10^6^ cells/ml were collected and washed with ice cold FACS buffer (PBS containing 0.5% BSA) and incubated with mouse monoclonal anti-FLAG M2 primary antibody (1:500) or rabbit polyclonal anti-T2R4 antibody (1:300, for detection of endogenous T2R4 in hASMCs) in FACS buffer for 1 h on ice. The cells were then washed 2 to 3 times with FACS buffer and incubated with Alexa 488 goat anti-mouse (1:1000) or goat anti-rabbit secondary antibody (1:1000) as shown previously [18, 23]. For analyzing chaperone activity of quinine, FLAG-tagged T2Rs were incubated with APC-conjugated anti-FLAG monoclonal antibody (1:500) for 1 h on ice in the dark. Following washing, cells were resuspended in 300 μl FACS buffer. The fluorescence signals of 1×10^4^ cells/tube were measured using single-color analysis by the BD FACS Canto. The results were analyzed using the FACS Diva and FlowJo software programs. The cell surface expression was calculated in terms of Mean Fluorescence Intensity (MFI) as percentage increase over control, i.e., expression of T2R4 without agonist stimulation for HEK293T cells, or untreated hASMCs, which was set at 100%. The TPα transfected cells were used as control because this receptor does not internalize upon stimulation with U46619 [24].

### Measurement of Intracellular Calcium

FLAG-T2Rs were expressed in HEK293T cells using 3μg DNA per 1×10^6^ cells in six-well tissue culture plates. After 6–8 h of transfection, 1×10^5^ cells/well were plated in 96-well black-walled clear bottom plates. After another 14–16 h, the cells were loaded with 100 μl/well calcium-sensitive Fluo-4 NW dye (Invitrogen) for 40 min at 37°C and another 30 min at room temperature. Probenecid, which inhibits organic-anion transporters located in the cell membrane, was added to the dye. This ensured that the calcium measured was from intracellular sources, not extracellular. Changes in intracellular calcium were measured after addition of quinine or yohimbine by FlexStation-3 microplate reader (Molecular devices), at 525 nm emission following excitation at 494 nm.

### Brefeldin A treatment

T2R4-expressing HEK293T cells were pretreated with 5 μg/ml Brefeldin A (BFA) for 4 h at 37°C and then incubated with 1 mM quinine for 15 min or 1 h. The reactions were stopped by keeping the cells on ice and then processed for cell surface expression analysis of T2R4 by flow cytometry as described previously.

### Desensitization assays

FLAG-T2R4-expressing HEK293T cells (1×10^5^ cells/well), or hASMCs, were plated in poly-L-lysine coated 96-well black-walled clear bottom plates. The cells were loaded with Fluo-4 NW dye for 40 min at 37°C followed by 30 min at room temperature. During the final 15 min, the cells were exposed to 1 mM quinine or yohimbine, in the presence of dye. This is represented as a pretreatment, whereas, T2R4-expressing cells without any quinine or yohimbine pretreatment, and treated with assay buffer (1×HBSS, 20 mM HEPES) only are denoted as untreated cells. The cells were washed twice with assay buffer after the pretreatment and the wells were loaded with 100 μl of dye again. Changes in intracellular calcium were measured for the next 3 min after 1 mM quinine or yohimbine was added by the FlexStation 3 microplate reader. Quinine-mediated Ca^2+^ responses of untreated cells, used as control, were considered as 100% and Ca^2+^ responses of pretreated cells were normalized using this value.

### Statistical analysis

All the statistical analyses were performed using GraphPad Prism version 5.0 software. Statistical analysis was performed using one-way analysis of variance (ANOVA) with *Tukey’s* or *Dunnette’s* post hoc test, two-way ANOVA with *Bonferroni’s* multiple comparison or unpaired two-tailed Student’s *t* test from a minimum of 3 independent experiments to determine the statistical significance wherever applicable. * p<0.05, ** p<0.01, *** p<0.001.

## Results

### Desensitization of T2R4

**Fig 1:**
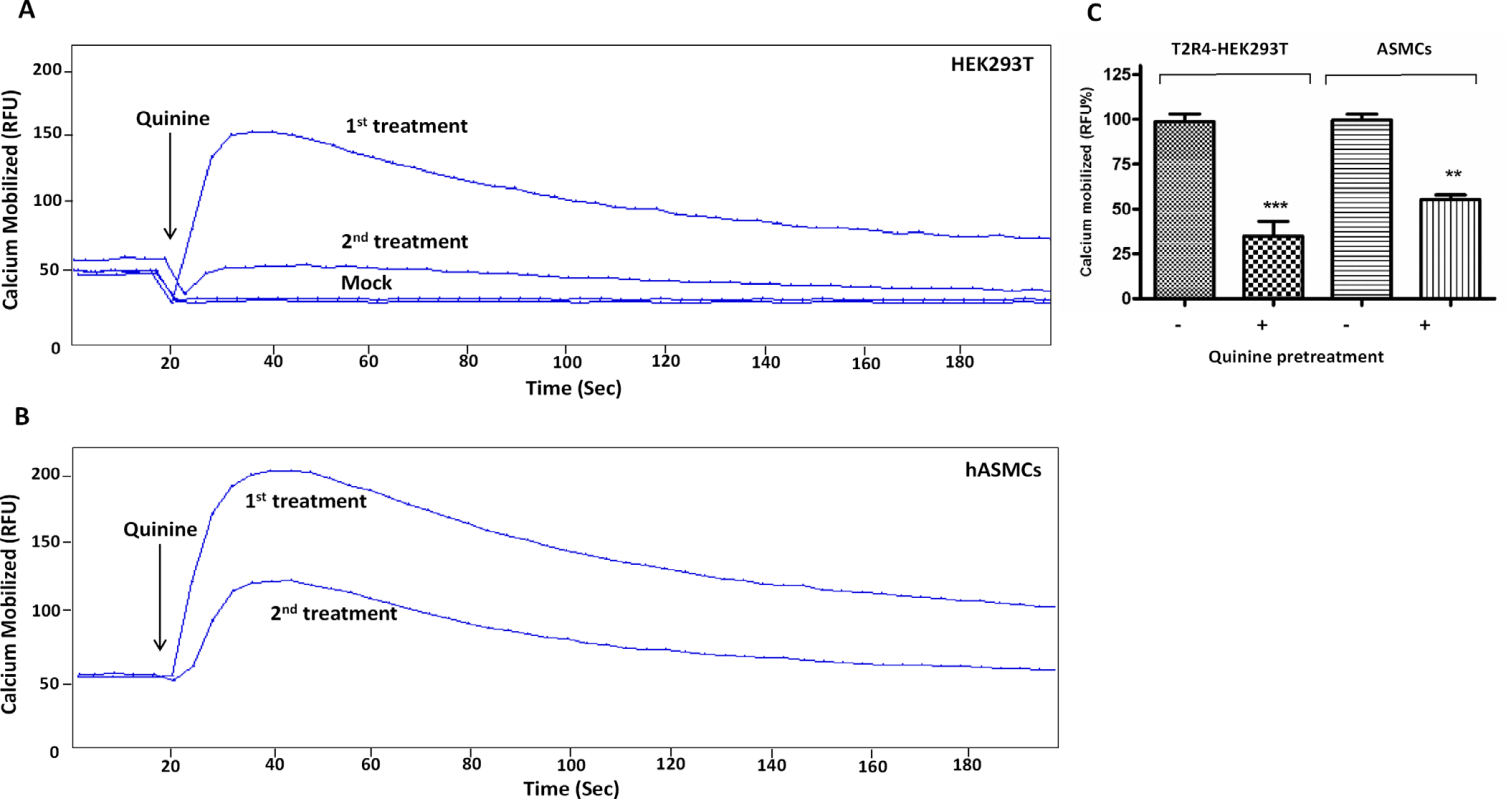
Quinine induced desensitization of T2Rs. Representative calcium traces showing desensitization of the quinine response to quinine pretreatment in HEK293T cells and hASMCs. **[A]**. Fluo-4 NW loaded T2R4 expressing HEK293T cells and mock-transfected HEK293T cells were pretreated with 1 mM quinine for 15 min, washed with assay buffer and re-exposed to 1 mM quinine. The intracellular calcium mobilized was measured in terms of relative fluorescence units (RFU) at 525 nm following excitation at 494 nm, using a FlexStation 3 fluorescence microplate reader. **[B]**. Human ASM cells pretreated with quinine for 15 min, washed with assay buffer and re-exposed to quinine were used as control. Cells pretreated with assay buffer and then exposed to quinine represent first treatment. Cells pretreated with quinine and re-exposed to same concentration of quinine represent second treatment. The arrow at 20 sec indicates the addition of agonist by Flexstation 3 microplate reader. **[C]**. Bar plot representation of the calcium responses in T2R4-HEK293T cells and hASMCs in response to quinine treatments. The results are represented with the calcium mobilized in untreated cells taken as 100%. Results are from a minimum of three independent experiments. Statistical significance was determined by student *t-*test.

GPCRs that undergo desensitization display reduced responsiveness to a second stimulation with the same agonist [25]. To examine T2R4 desensitization, T2R4 expressing HEK293T cells were pretreated with the agonist quinine for 15 min, after which they were washed with assay buffer before re-exposure to the same concentration (1 mM) of quinine. Changes in intracellular calcium were measured for the next 3 min. Quinine pretreatment led to a decrease in subsequent quinine-mediated calcium responses to 35 ± 5% (equivalent to 60% ± 5.2% desensitization) compared to the control or untreated cells (Fig 1A and 1C). Quinine induced desensitization of endogenous T2Rs was examined in human ASMCs previously [14], so this was used as an external control in our study. In our experimental conditions, quinine pretreatment of ASMCs resulted in reduction of calcium response to 55% of the control (equivalent to 45% ± 2.8% desensitization of T2Rs, Fig 1B and 1C).

**Fig 2:**
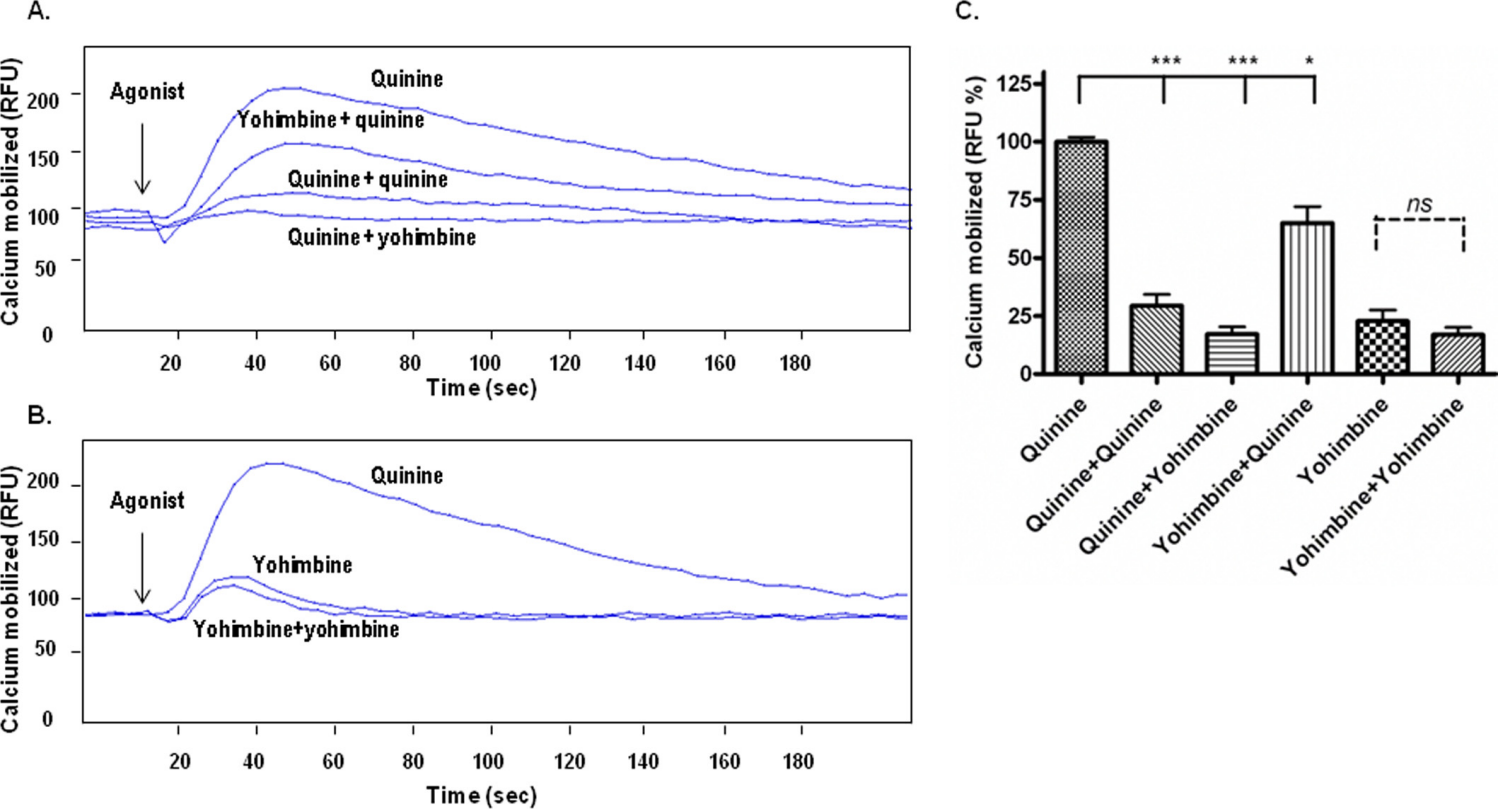
Agonist promoted desensitization of T2R4 expressed in HEK293T cells. **[A]**. Representative calcium traces of T2R4-expressing HEK293T cells pretreated with assay buffer, quinine or yohimbine. **[B]**. Representative calcium traces of T2R4-expressing HEK293T cells pretreated with assay buffer or yohimbine. Fluo-4 NW loaded cells were pretreated with buffer alone or 1 mM each of quinine or yohimbine for 15 min, washed with assay buffer, and exposed to same concentrations of the agonist by the Flexstation 3 microplate reader. Changes in intracellular calcium were measured in terms of relative fluorescence units (RFU) at 525 nm following excitation at 494 nm. The arrow at 20 sec indicates the addition of agonist by Flexstation 3 microplate reader. **[C]**. Bar plot representation of the measured intracellular calcium. Buffer pretreated T2R4-expressing cells stimulated with quinine were represented as 100%, and calcium responses of other treatments were normalized to it. Data is representative of four independent experiments and expressed as mean ± SEM. Significance was checked by one-way ANOVA using *Dunnette’s* post hoc test, *p<0.05, ***p<0.001.

Additional studies for T2R4 desensitization were performed by using another structurally distinct T2R4 agonist, yohimbine [2, 19]. Pretreatment with 1 mM yohimbine evoked only a marginal desensitization of the subsequent yohimbine-induced calcium responses (Fig 2B and 2C). We checked cross-compound desensitization and noticed that quinine pretreatment evoked desensitization of the subsequent yohimbine induced calcium responses amounting to 17 ± 3% (Fig 2A and 2C). However, T2R4-expressing cells pretreated with yohimbine and subsequently exposed to quinine displayed calcium responses amounting to 65 ± 7%, thus displaying reduced desensitization of the receptor (Fig 2A and 2C).

### Agonist-mediated T2R4 trafficking

**Fig 3:**
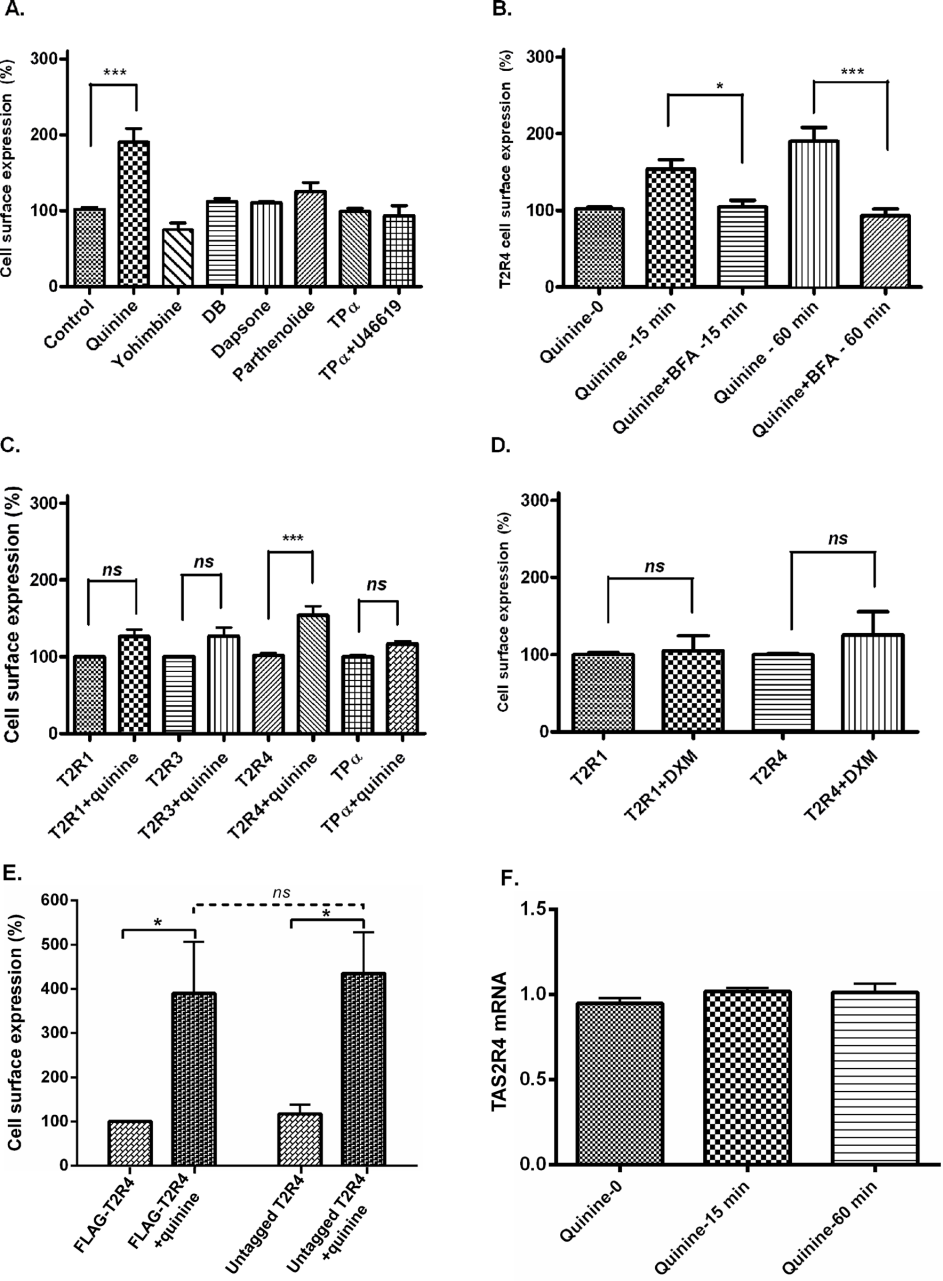
T2R trafficking and chaperone activity of quinine. [**A**]. Effect of agonist treatment on T2R4 cell surface expression. T2R4-HEK293T cells were treated with buffer (control) or 1 mM each of quinine, yohimbine, dapsone and parthenolide, or 2 mM denatonium benzoate (DB) for 1 h at 37°C. TPα expressing HEK293T cells treated with its agonist, 1 μM U46619, were used as a positive control. [**B**]. Effect of BFA treatment on T2R4 trafficking. T2R4 expressing HEK293T cells were treated with vehicle or 5 μg/ml BFA for 4 h at 37°C. The cells were incubated with 1 mM quinine, in the presence of BFA, for 15 min or 60 min at 37°C. Cell surface expression of T2R4 was determined by flow cytometry and expressed as percentage increase over FLAG-T2R4 without quinine stimulation (basal, represented as quinine-0), which was considered as 100%. Values represent the mean ± SEM of 3–7 independent experiments. [**C**]. Effect of quinine treatment on cell surface expression of FLAG-tagged T2R1, T2R3, T2R4 and TPα transiently expressed in HEK293T cells. [**D**]. Effect of DXM treatment on surface expression of transiently expressed T2R1 and T2R4 in HEK293T cells. Receptor-expressing cells were incubated with 500 μM DXM for 15 min before determining cell surface expression by flow cytometry. Receptor expression was analyzed using mouse monoclonal anti-FLAG antibody, which was detected by goat anti-mouse Alexa 488 antibody. Significance was checked by one-way ANOVA using *Tukey’s* post test or by Student’s *t-test*. **[E].** Effect of FLAG sequence on T2R4 internalization or expression. HEK293T cells expressing either FLAG-T2R4 or untagged T2R4 were treated with buffer (control) or 1 mM quinine for 1 hr at 37°C. Cell surface expression of T2R4 was determined by flow cytometry using polyclonal anti-T2R4 antibody targeting the extracellular surface of T2R4. Data was represented as percentage change over FLAG-T2R4 without quinine stimulation (represented as untreated), which was considered as 100%. Values represent the mean ± SEM of 3–4 independent experiments performed in duplicates (Mean ± SEM). Statistical significance was checked by Two-way ANOVA using *Bonferroni’s* multiple comparison test. * *p* <0.05. [F]. Effect of quinine on TAS2R4 mRNA levels. Serum-starved TAS2R4 expressing HEK293T cells were treated either with assay buffer or 1 mM quinine for 15 min or 60 min. Total RNA was extracted and reverse transcribed into cDNA. TAS2R4 expression was examined by performing real-time PCR using TAS2R4-specific primers. The mRNA expression is shown as fold change over TAS2R4 without any quinine stimulation. Data are mean ± SEM from three independent experiments.

To examine whether agonist stimulation leads to T2R4 internalization, cell surface expression of T2R4 was examined by flow cytometry, with and without agonist treatment. We selected bitter compounds that were shown to activate T2R4; quinine, yohimbine, denatonium benzoate (DB), dapsone and parthenolide [2]. The respective concentrations were selected based on their published threshold values, and keeping in view the low affinity of T2Rs for their ligands, usually in higher micromolar to millimolar concentrations [2, 9]. T2R4 expressing cells were incubated with the compounds for 1 h at 37°C. N-terminal FLAG-tagged TPα, which does not internalize upon stimulation with its agonist, U46619, was used as a positive control. None of the compounds, except quinine, caused any significant change in T2R4 cell surface expression (Fig 3A). Interestingly, quinine upregulated the cell surface expression of T2R4 as demonstrated by flow cytometry analysis of the FLAG-tagged T2R4. DB, yohimbine, dapsone and parthenolide did not cause any statistically significant increase in T2R4 expression. Quinine caused a two-fold increase in T2R4 cell surface expression with a statistical significance of ***p<0.001.

To elucidate whether quinine-induced upregulation of T2R4 expression was a post-translational event, we used BFA which is an inhibitor of protein transport from the endoplasmic reticulum (ER) to the Golgi, and affects trafficking of the protein to plasma membrane. T2R4-expressing cells were treated with 5 μg/ml BFA for 4 h and then exposed to quinine or vehicle for 15 min or 60 min, in the presence of BFA. This BFA treatment completely inhibited the trafficking of T2R4 by quinine and the expression levels were comparable to that of untreated control (Fig 3B).

### Chaperone activity of quinine

We wanted to investigate if the observed chaperone action of quinine was T2R4-specific and/or agonist-specific. Therefore, we selected three more GPCRs, T2R1, T2R3 and a Class A GPCR, TPα, for further studies. A 15 min treatment of quinine did not produce any significant change in surface expression of T2R1, T2R3 and TPα (Fig 3C). In contrast, surface expression of T2R4 increased 1.5 fold in response to quinine. Quinine is a membrane-permeant molecule which can interact with other intracellular proteins [26]. To determine if other amphiphilic bitter compounds are capable of producing the same effect as quinine, we selected dextromethorphan (DXM), a membrane-permeable agonist of T2R1 [20, 27, 28]. Cells expressing T2R1 or T2R4 were exposed to DXM for 15 min before surface expression was analyzed by flow cytometry. DXM treatment caused no significant increase in surface expression of T2R1 or T2R4 (Fig 3D). The data thus suggests that the increased surface expression of T2R4 produced by quinine is both receptor and agonist-specific.

To test whether the FLAG-tag somehow inhibits the internalization process, we pursued experiments using an untagged T2R4. The results showed no significant difference between FLAG-T2R4 and untagged T2R4 cell surface expression upon quinine treatment (Fig 3E). Further, to elucidate whether quinine-induced upregulation of T2R4 expression was due to a gene transcription event, real-time PCR analysis was pursued to analyze changes in the TAS2R4 transcript after 15 min or 60 min quinine treatment. No increase in TAS2R4 mRNA levels was observed (Fig 3F).

**Fig 4:**
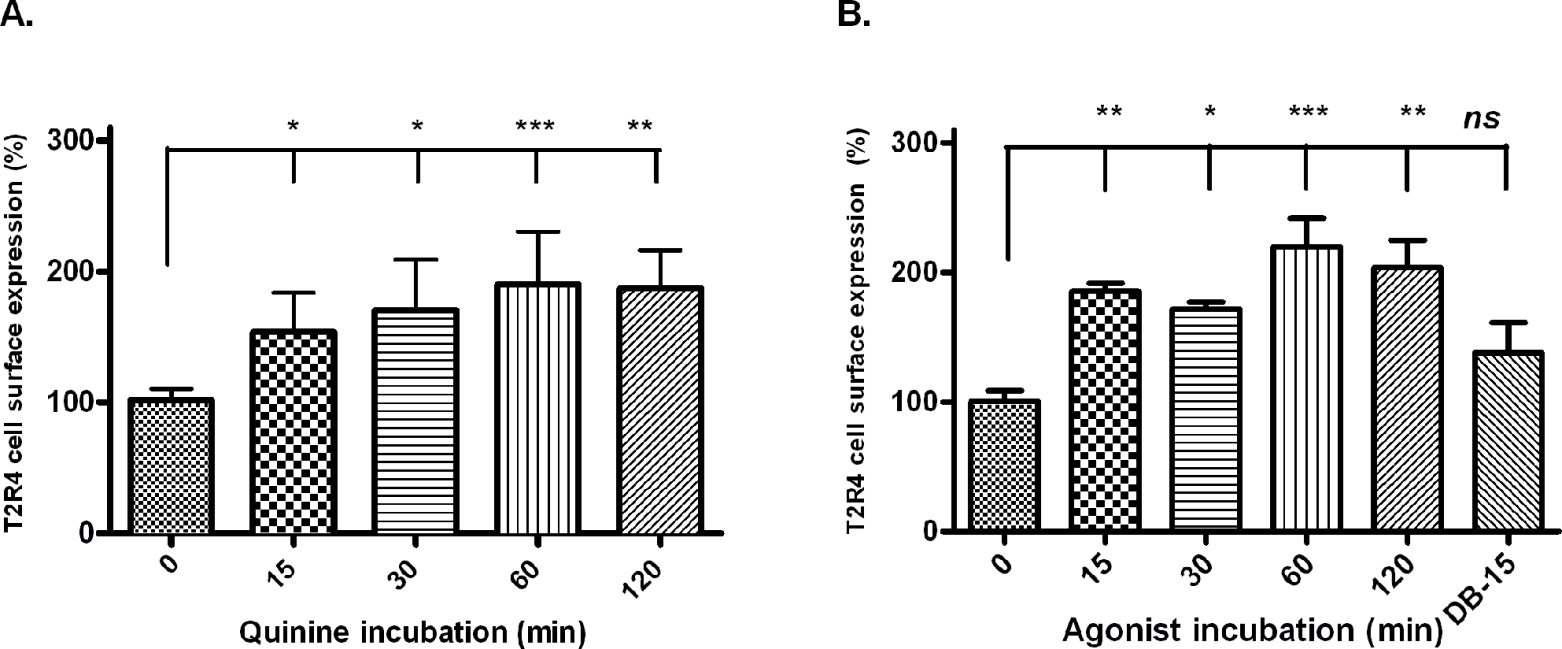
Time course studies of quinine incubation times on T2R4 surface expression. [**A**]. Time-dependent effect of quinine incubation on cell surface expression of T2R4 in HEK293T cells. T2R4 expressing cells were treated with quinine for 15 min, 30 min, 60 min or 120 min at 37°C. T2R4 cell surface expression was analyzed by flow cytometry using mouse monoclonal anti-FLAG antibody. [**B**]. Time-dependent effect of quinine incubation on surface expression of endogenous T2R4 in hASMCs. T2R4 surface expression was also determined after denatonium benzoate (DB) incubation for 15 min, which produced no change in surface expression of T2R4 in hASMCs. Data represents values from three independent experiments performed in duplicates. Significance was checked by one-way ANOVA using *Tukey’s* post hoc test, *p<0.05, **p<0.01, ***p<0.001.

We then examined whether the effects of quinine on receptor expression varied with agonist incubation time. For this, T2R4 expressing cells were incubated with quinine for 15 min, 30 min, 60 min and 120 min at 37°C before receptor expression was analyzed. Untreated T2R4 was used as control. The cell surface expression of T2R4 increased in a time-dependent manner, reaching the peak at 60 min incubation and then stabilizing (Fig 4A). The expression increased from 1.5-fold at 15 min incubation to almost 2-fold at 60 min quinine incubation. Human ASMCs were used as a control in desensitization assays, therefore, we assessed the effect of quinine on surface expression of endogenous T2R4 in these cells. Surface expression of T2R4 in hASMCs increased in a time-dependent manner in response to quinine (Fig 4B). This data was consistent with the flow cytometry data of T2R4 transiently expressed in HEK293T cells.

**Fig 5:**
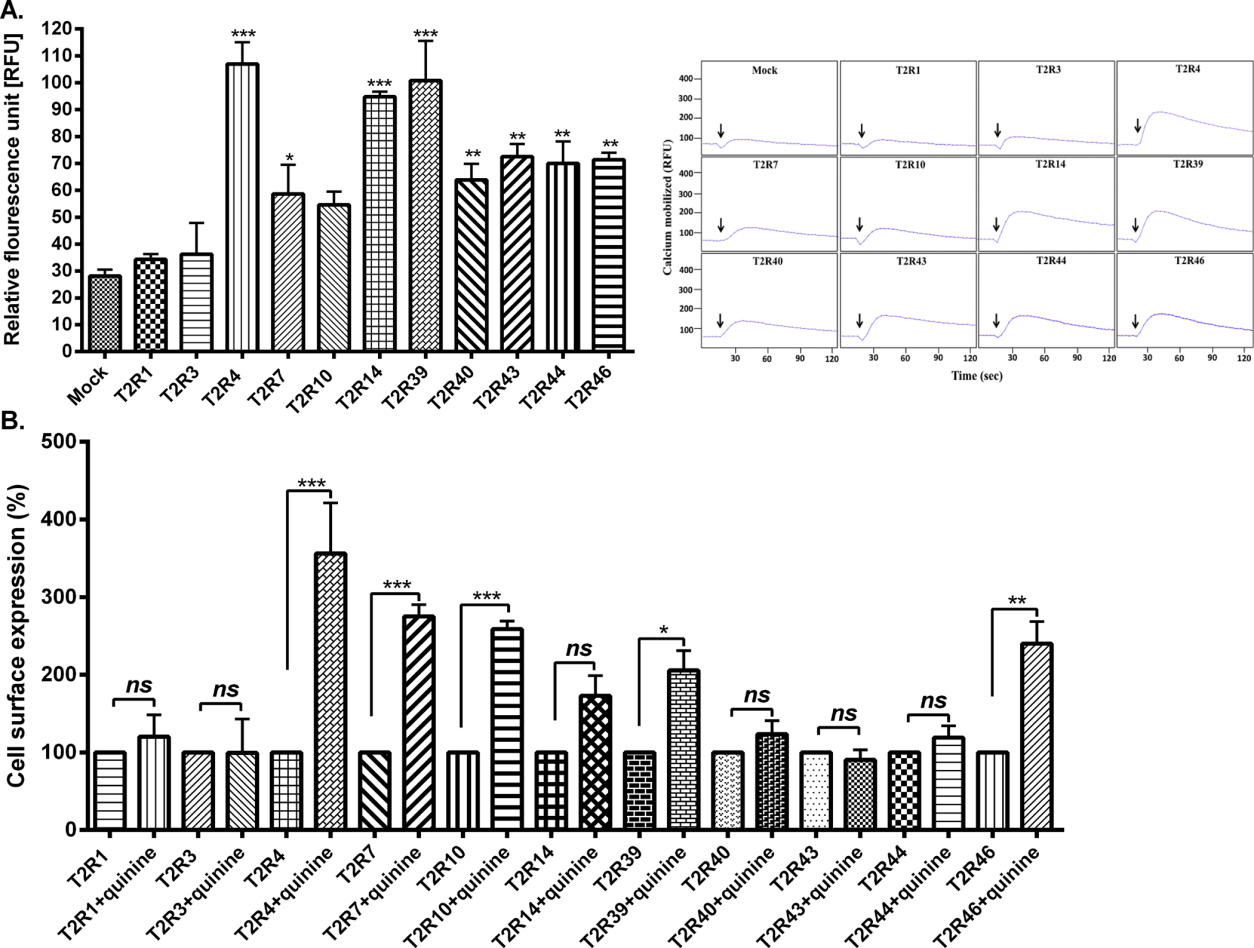
Agonist and chaperone activity of quinine on T2Rs. **[A].** Quinine agonistic activity on 11 T2Rs (left panel). HEK293T cells expressing the 11 T2Rs and mock-transfected control (pcDNA3.1 vector alone) were treated with 1 mM quinine and calcium responses were recorded as relative fluorescence unit (RFU). Representative calcium traces of quinine response for different T2Rs expressed in HEK293T cells and mock-transfected cells (control) were presented in the right panel. Data represents values from at least three independent experiments performed in triplicates. Significance was checked by one-way ANOVA using *Dunnet’s* post hoc test, *p<0.05, **p<0.01, ***p<0.001. **[B].** Trafficking and chaperone activity of quinine on 11 T2Rs. HEK293T cells expressing FLAG-T2Rs were incubated with buffer or 1 mM quinine for 1 hr at 37°C. The cells were stained with FLAG-antibody (1:500 dilution) for 1 hr on ice in the dark and washed with FACS buffer for 2–3 times. Flow cytometry analysis was performed to study the effect of quinine on cell surface expression of T2Rs. The MFI values of the mock-transfected cells were subtracted from both treated and untreated cells expressing the respective T2Rs. The data was then normalized to untreated cells taken as 100% for each T2R. Data represents values from at least three independent experiments performed in duplicates. Significance was checked by Student t-test, *p<0.05, **p<0.01, ***p<0.001.

Along with T2R4, quinine was reported to activate T2R7, T2R10, T2R14, T2R39, T2R40, T2R43, T2R44 and T2R46 at a threshold concentration of 10 μM [2]. Since there are no reports on the EC_50_ value of quinine for these T2Rs, we performed calcium mobilization assays using the EC_50_ value of quinine for T2R4 (1 mM). T2R14 and T2R39 showed a significant response equal to T2R4 while T2R7, T2R40, T2R43, T2R44 and T2R46 showed low to moderate response. T2R10 showed no significant response with 1 mM quinine treatment compared to mock-transfected control cells (Fig 5A). T2R1 and T2R3 which are not known to be activated by quinine were also included as external controls (Fig 5A). Next to test the chaperone activity of quinine on these T2Rs, a 1 h quinine (1 mM) treatment followed by FACS analysis was pursued. The MFI values of the mock-transfected cells were subtracted from both treated and untreated cells.

Cell surface expression from treated cells of each receptor was normalized to their respective untreated cells. Significant increase in the cell surface expression of T2R4, T2R7, T2R10, T2R39 and T2R46 was observed upon quinine treatment. No significant change in expression of T2R14, T2R40, T2R43 and T2R44 was observed (Fig 5B). Strikingly, none of the T2Rs internalized under the conditions tested.

## Discussion

In this study, we demonstrate that quinine causes 60% ± 5.2% desensitization of T2R4 expressed in HEK293T cells, while it causes 45% ± 2.8% desensitization of endogenous T2Rs in hASMCs (Fig 1). This slight discrepancy can be attributed to the ability of quinine to activate nine T2Rs with different efficacies [2], which might be the case in hASMCs. However, yohimbine pre-treated T2R4 displayed 20 ± 3% desensitization to subsequent yohimbine exposure (Fig 2B and 2C). Cross-compound desensitization was evident as quinine pretreated T2R4 evoked 78 ± 3% desensitization of the subsequent yohimbine response. In contrast, pre-treatment with yohimbine showed 35 ± 7% desensitization of the receptor to subsequent quinine response. The differential desensitization profile of T2R4 to its two structurally distinct agonists can be speculated in terms of biased agonism. This concept has not been explored for T2R agonists but biased ligands have been identified for many GPCRs. A striking example of ligand biased regulation of desensitization and endocytosis is provided by the μ-opioid receptor [29]. Methadone and buprenorphine were shown to have desensitization properties different from those of morphine for μ-opioid receptor [29]. Similarly, the recovery from prolonged activation of 5-HT_3_ receptors is differential among partial agonists and full agonists [30]. It remains to be investigated if quinine and/or yohimbine are full agonists or partial agonists for T2R4.

Desensitization of GPCRs is initiated when their cytosolic residues, containing either serine or threonine, are phosphorylated by selected kinases [12]. In T2R4, there are 13 serine and threonine residues on the intracellular side which are not well conserved among the 25 human T2Rs [16, 18]. Among these, a few threonines in the third intracellular loop and the C-terminus are involved in T2R4 expression and/or function [16, 18]. It is well documented that most GPCRs in response to agonist undergo phosphorylation in the third intracellular loop or the cytoplasmic tail. Given that a number of the non-conserved serine and threonine residues in T2R4 can be potentially phosphorylated and might also be involved in receptor expression and function [16, 18], currently it is not feasible for us to delineate all these effects. Therefore, studies directed at elucidating the T2R4 residues involved in phosphorylation could not be pursued at this time.

Arrestin binding to the phosphorylated GPCRs uncouples them from the heterotrimeric G proteins, and may also initiate receptor internalization [31]. However, in addition to these regulatory pathways, some GPCRs undergo pharmacological sequestration, which is desensitization without their actual internalization into the intracellular compartments [32]. These receptors may be sequestered on or near the plasma membrane without internalizing, either as insulation on the membrane [33] or via conformational changes of receptor proteins [32], thus rendering them inaccessible to their agonists. We expected quinine-incubation to cause T2R4 internalization. In contrast, quinine treatment caused an increase in cell surface trafficking of T2R4 in a time-dependent manner (Fig 4). However, other bitter compounds previously shown to activate T2R4, yohimbine, DB, dapsone and parthenolide did not cause any significant increase in T2R4 cell surface trafficking (Fig 3A). Interestingly, these compounds did not lead to receptor internalization either.

To assess the cause of increased T2R4 expression, we analyzed if quinine was causing any transcriptional changes. However, q-PCR data revealed no significant changes in the mRNA levels of TAS2R4 after treatment with quinine for 15 min or 60 min (Fig 3E). These data, argue against the contribution of a gene transcriptional event in quinine-induced upregulation of T2R4 expression. Next, we analyzed if quinine was causing any post-translational changes. Quinine incubation, in the presence of BFA, revealed that quinine-induced translocation of T2R4 to the plasma membrane was BFA-sensitive. Following synthesis in the ER, correctly folded GPCRs traffic to the Golgi and are targeted to the plasma membrane in their mature form. ER functions as a quality control system in cells which retains the misfolded proteins for degradation [34]. By disrupting the Golgi apparatus, BFA interrupts maturation and membrane targeting of the receptor. BFA treatment for 4 h inhibited receptor trafficking to the cell surface and showed expression of T2R4 comparable to that of control (Fig 3B). This data might be suggestive that only 50% of the synthesized T2Rs reach the mature form, and are targeted to the plasma membrane under basal conditions. GPCRs, except rhodopsin, are expressed at very low levels in native systems [35]. Different strategies, like codon optimization and use of export tags at N-terminus, have been used to enhance the expression of T2Rs in heterologous systems [6, 22]. Our results suggest that the short FLAG affinity sequence at the N-terminus does not interfere with T2R4 internalization or expression (Fig 3E). Taken together, our results suggest that quinine acts intracellularly to enhance T2R4 expression and thus acts as a pharmacological chaperone for T2R4.

The chaperone-like effects of agonists and antagonists have been previously reported where they can stabilize the native conformation of GPCRs and facilitate their export from ER to plasma membrane and protein maturation [36, 37]. Such functional rescue suggests that pharmacological chaperones represent novel therapeutic agents for the treatment of conformational diseases [36, 38, 39]. It raises the question as to why the other T2R4 agonists tested did not result in an upregulation of its expression (Fig 3). Studies report that nonpeptide ligands behave as pharmacological chaperones to facilitate anterograde trafficking of the receptor along the biosynthesis pathway, indicating that membrane permeability is required for the chaperone roles [37]. Thus the agonist, like quinine, should be cell-permeable [26] to act intracellularly and affect biosynthesis of the protein to produce chaperone effects. However, as data from this study indicates, this chaperone activity is both receptor and agonist-specific. In addition to its antimalarial action, quinine is capable of eliciting bronchodilation in asthmatic models [10, 11]. Chemical chaperones have been shown to have therapeutic potential in the treatment of asthma [40].

As quinine is not specific to T2R4, we extended our study to investigate the chaperone activity on other T2Rs known to be activated by quinine, namely T2R7, T2R10, T2R14, T2R39, T2R40, T2R43, T2R44 and T2R46 [2]. Results from our calcium mobilization assays suggest that except for T2R10, others that include T2R7, T2R14, T2R39, T2R40, T2R43, T2R44 and T2R46 show significant activation with 1 mM quinine (Fig 5A). FACS analysis of the cell surface expression of these T2Rs revealed the chaperone activity of quinine for T2R7, T2R10, T2R39 and T2R46 (Fig 5B). Comparison of the calcium mobilization with FACS analysis (Fig 5A and 5B) shows no correlation between agonist activities of quinine with its chaperone activity. For example, T2R14, T2R40, T2R43, T2R44 that are significantly activated by quinine, display no increase in cell surface expression upon 1hr treatment with quinine. On the other hand, T2R10 which is not activated by quinine showed significant increase in cell surface expression upon quinine treatment. These results suggest that the agonistic activity and chaperone activity are independent for quinine.

Recent studies demonstrate that residues involved in GPCR desensitization and internalization do not strictly overlap [41–43]. Agonists like morphine produce desensitization of mu-opioid receptors without their significant internalization [44]. Our data supports this emerging evidence that GPCR desensitization and internalization are significantly independent processes. The current study reports for the first time a unique pharmacochaperone activity for a common bitter ligand. Quinine is an anti-malarial and bitter tasting compound which is also used to treat rheumatoid arthritis and lupus [45]. Given that T2Rs are expressed in multiple tissues, and the novel chaperone activity of quinine, more studies are needed to elucidate the clinical relevance of these findings in physiological and pathophysiological states involving T2R signaling. We conclude from our results that T2Rs do not get internalized upon agonist stimulation, instead, quinine positively influences T2R4, T2R7, T2R10, T2R39 and T2R46 trafficking by acting as a pharmacological chaperone. This study provides novel insights into the molecular mechanisms leading to human bitter taste signal termination.
